# Job embeddedness and missed nursing care at the operating theatres: the mediating role of polychronicity

**DOI:** 10.1186/s12912-023-01628-8

**Published:** 2023-12-04

**Authors:** Ahmed Abdelwahab Ibrahim El-Sayed, Maha Gamal Ramadan Asal, Rabab Saleh Shaheen, Sally Mohammed Farghaly Abdelaliem

**Affiliations:** 1 Nursing Administration Department, Faculty of Nursing, Alexandria University, Alexandria, Egypt; 2 Medical Surgical Nursing Department, Faculty of Nursing, Alexandria University, Alexandria, Egypt

**Keywords:** Job embeddedness, Missed care, Nurses, Nursing care, Operating theatre, Polychronicity

## Abstract

**Background:**

Perioperative missed nursing care is a serious issue that can compromise patient safety and quality of care. However, little is known about the factors that influence perioperative missed nursing care.

**Aim:**

This study aimed to examine the effects of job embeddedness and polychronicity on perioperative missed nursing care as well as to test the mediating role of polychronicity on the relationship between job embeddeness and perioperative missed nursing care.

**Method:**

This was a cross-sectional correlational study that used a convenience sample of 210 operating room nurses from nine hospitals in Egypt. Data were collected using self-administered questionnaires that measured job embeddedness, polychronicity, and perioperative missed nursing care. Structural equation modeling was used to test the hypothesized relationships among the variables.

**Results:**

The findings demonstrated a significant negative and moderate association between missed perioperative care and both nurses’ job embeddedness and polychronicity. Moreover, there was a moderately positive and significant correlation between polychronicity and job embeddedness. Path analysis revealed a significant positive causal effect between job embeddedness and polychronicity. The results of mediation revealed that the indirect effect of job embeddedness on missed care through polychronicity was statistically significant; suggesting that polychronicity partially mediated this relationship.

**Conclusion:**

This study sheds light on the intricate relationship between nurses’ job embeddedness, missed care, and polychronicity in the operating theater context. By enhancing job embeddedness and fostering polychronicity among nurses, healthcare organizations can reduce perioperative missed care and ultimately improve patient care outcomes in this critical healthcare setting.

**Supplementary Information:**

The online version contains supplementary material available at 10.1186/s12912-023-01628-8.

## Introduction

In today’s healthcare system, nurses play a critical role in ensuring that patients receive the highest quality care possible. Their role is particularly crucial in the operating theater, where the preparation, execution, and recovery phases of surgery require a significant amount of attention and precision [1]. However, due to various contextual factors, such as workload and time constraints, nurses may experience barriers to the provision of care, resulting in missed care [2]. Missed care is defined as a failure to complete essential care actions for patients, and this can include anything from not administering medications to not performing assessments or vital sign checks [3]. It has been well-documented that missed care contributes to negative patient outcomes, longer hospital stays, and higher costs. Missed nursing care (MNC) is a worldwide concern that jeopardizes the patient’s wellbeing and safety [4]. Throughout the world, between 10% and 50% of nursing care is neglected [5].

Missed perioperative nursing care refers to situations where aspects of nursing care that should have been provided are inadvertently overlooked or not completed as intended [6]. Such occurrences can arise from a variety of factors, including staffing issues, communication breakdowns, time constraints, and individual oversight [7]. In regards to the operating room (OR), missed care can be particularly impactful. The OR is a dynamic environment with numerous moving parts, making it susceptible to occasional oversight [8]. The OR environment presents unique challenges that can affect nurses’ ability to provide optimal care. Key aspects of nursing care that may be missed in this setting include proper patient positioning, preoperative medication administration, timely documentation, and effective handovers between healthcare professionals [9].

One example of missed care in the OR is insufficient attention to infection control protocols [10]. In the pursuit of expeditious turnover times, disinfection procedures or the proper sterilization of equipment may be inadvertently overlooked. This oversight increases the risk of surgical site infections, posing a threat to patient safety and recovery [11]. Another common occurrence is the failure to adequately address patients’ psychological and emotional needs during the perioperative period. The OR can be an anxiety-inducing environment, and patients may benefit from soothing interventions such as preoperative education, reassurance, and emotional support. When these aspects of care are overlooked, patients may experience increased anxiety, leading to potential negative effects on their surgical outcomes [12].

To prevent and mitigate the occurrence of missed care, several strategies can be employed. First and foremost, fostering a culture of open communication and collaboration among healthcare professionals is crucial [13]. Encouraging interdisciplinary team meetings, debriefings, and regular audits can help identify areas of improvement and facilitate better coordination [14]. Additionally, implementing standardized protocols and checklists can serve as valuable tools to ensure that essential care is not missed. These protocols can outline specific steps in the perioperative process, allowing for consistent and comprehensive care delivery [3].

One potential factor that can influence nurses’ ability to complete their work without omissions is their level of job embeddedness (JE). JE was defined as the degree to which nurses are engaged, affiliated, and attached to their work [15]. JE is a set of psychological, personal, and professional characteristics that keep nurses from leaving their jobs [16]. JE is considered a better predictor of nurse job outcomes such as attendance, performance, and retention. It also impacts nurses’ commitment to their hospital, which increases teamwork, cooperation, emotional bonds, and a desire to expand the organization [17].

JE integrates three aspects: link, fit, and sacrifices [18]. Link describes a nurse’s relations and connection with others, participation in decision-making, and involvement on work team. Fit refers to a nurse’s close connection to the organization and professional commitment. It relies on nurses’ alignment with the organization’s culture, philosophy, job demands, and career goals [19]. Finally, sacrifice describes nurses’ perceived sacrifices when they leave hospitals [20]. Porter et al. (2019) highlighted that sacrifice in the context of JE as giving up something valued in favor of other concerns [21]. Salary, pension fund, paid time off, sick leave, and other additional benefits are all examples of sacrifices. Additionally, they can be subtle psychological losses, such as office space, losing seniority, parking privileges, promotion chances, job stability and loss of further knowledge and experience that shape nurses’ career aspiration [22].

Studies have found that embedded employees spend considerable times in their organizations. Additionally, embedded employees serve as advocators for their organizations that help organizations to remain sustainable and competitive [17]. JE fosters positive practices among employees such as sickness presenteeism, green workplace practices, and the holism approach [23, 24]. Meanwhile, nurses who are embedded in their jobs are less likely to postpone critical tasks, and engage in counterproductive behaviors and incivility [25]. Embedded nurses exhibit high innovative work behavior and have high tolerance for unplanned change and uncertainty [15]. Job embedded nurses are productive and able to provide value added suggestions to improve their organizations and their practice. They seek perfectionism in their work which guarantees that basic tasks are accomplished in an appropriate manner [26].

Most previous scientific work has addressed JE among nurses in the context of critical care; however, limited studies have addressed this construct in the context of ORs. Additionally, the nursing literature places much emphasis on the impacts of JE on nurses’ outcomes such as turnover, job satisfaction, and nurses’ performance. In addition, prior studies [20, 27, 28] revealed that JE could contribute to decreasing omissions, and close calls for patient care; however, the exact role of JE in reducing MNC, especially in the realm of perioperative care, is not clear.

The relationship between nurses’ JE and missed perioperative nursing care is complex and may be influenced by various factors. Polychronicity is a critical factor that has received high momentum in nursing the literature in terms of its ability to shape nursing and patient care outcomes and may exert a significant role if it interacts with other variables, including JE, in reducing MNC [29]. Polychronicity refers to the ability to handle and manage multiple tasks at the same time. Polychronic individuals tend to multitask and are comfortable with interruptions and frequent shifts in focus, whereas, monochronic individuals tend to prefer sequential tasks and a linear workflow. In the context of the operating theater, polychronicity may enable nurses to manage a high workload and prioritize tasks, reducing the likelihood of missed care [30].

Recent workplace changes including the application of information technology, career advancement, collaborative work, and downsizing. Meanwhile, the circumstances and speed of job performance are rapidly evolving, and the conventional concepts of job performance have become outdated for nursing [31]. These factors enhance the urge to augment polychronic behavior among nurses, especially in the context of ORs with high dynamic turbulent environments. In this respect, Some authors have recently claimed that nursing practice necessitates at least some degree of multitasking [32].

### Significance of the study and research gap

Previous works identified the types of MNS in different contexts as critical care [33], general medical surgical wards [34]; and nursing homes [35]. However, MNC is not adequately delineated in terms of its amount and types in operating theatres context. Our study addresses this gap and tries to examine the amount and types of missed perioperative nursing care. Moreover, previous studies examined sources of MNC in general medical wards, pediatric units, and obstetric units focusing on factors as job satisfaction [36], workload and staffing level [37], leader style and organizational culture [38], communication and teamwork [39] and supportive environment [40]. JE and polychronicity as critical variables in nursing practice received little consideration in nursing studies with limited studies examining the role of these factors in reducing the magnitude of missed nursing care especially in highly dynamic stressful atmosphere like operating theaters. Our study addresses this gap and examines the effect of both JE and polychronicity on missed perioperative nursing care.

In addition, different studies examined the relationship between JE and polychronicity in different context like educational settings [41], libraries, tourism [42] and business companies [43] with limited studies examined this relationship in nursing context. Furthermore, examining the interplay between missed perioperative nursing care, polychronicity and JE is of paramount importance for nursing practice as polychronicity and JE are significant attributes that shape nurses’ performance in different contexts, hence these factors exert valuable role in mitigating missed nursing role. Despite the promising role of examining this interplay in upgrading nursing performance and quality of care, no available studies tried to assess this interplay in operating theaters context. Our study addresses this gap and examines this interplay in the context of operating theaters.

### Theoretical Framework and Hypothesis Formulation

Our study is based on assumptions of reasoned action theory [44] and congruence model of organizational behaviour [45]. The theory of reasoned action suggested that when individuals decide whether to operate monochronically or polychronically, their choice depends on the tasks to be performed, the priorities assigned to the tasks, and their beliefs about how the tasks may be combined to the greatest advantage. Also, individuals choose to act polychronically if they perceive the task could be mastered efficiently in single time frame and this perception is fostered when the person find the job dimensions matches with his/her capabilities. In other words, the reasoned action theory assumed that when there is fit between the person and the job which reflect the concept of JE, the person often act polychronically. This means JE augment the polychronic tendency among individuals. According to congruence model, the higher congruence of person with his job makes him feel embedded and act polychronically, the result is exemplary performance. Thus, if JE (input) is experienced by the person acting polychronically (process), the resultant is high performance (output). This could explain why JE and polychronicity lead to reducing the likelihood of MNC. We developed a conceptual framework for our study based on the propositions of reasoned action theory and the directional predictives of congruence model. Accordingly, we suggested the following hypotheses:

H1: nurses’ JE will be significantly, positively associated with the polychronicity of OR nurses.

H2: nurses’ JE will be significantly and negatively associated with the perceived missed care at operating theatres.

H3: polychronicity will be significantly, negatively associated with missed care at operating theatres.

H4: polychronicity will mediate the associations between JE and missed care at operating theatres.

**Fig. 1:**
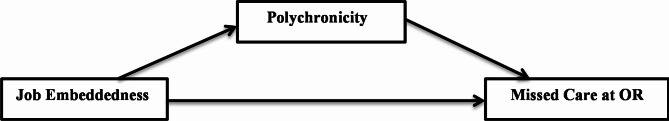
Conceptual Model Illustrating the Relationship between the Study Variables

Fig. 1 shows our proposed conceptual model illustrating the relationship between JE, missed perioperative nursing care, and polychronicity.

### Aim of the study

Our study aimed to investigate the mediating role of polychronicity in the relationship between nurses’ JE and MNC in the operating theatres. The objectives of our study are threefold. First, we aim to assess the amount and types of MNC at operating theatres. Second, we aim to identify the relationship between nurses’ JE and MNC at operating theatres. Third, we aim to investigate the mediating role of polychronicity in this relationship.

## Materials and Method

### Design and Participants

This is a multi-site, descriptive and cross-sectional study. This study was conducted in the operating theaters of nine university hospitals at Alexandria Governorate in Egypt. The study sample included OR nurses who worked in the study hospitals and agreed to participate in the study.

Using G*power (version 3.1.9.7), we determined that 325 nurses were required to achieve 0.8 power for effect size of 0.03, a type 1 error of 0.05 and two predictors. The questionnaire was distributed to all of the OR nurses of the study setting (n = 360) to account for the 10% attrition rate. A total of 341 OR nurses completed the questionnaire, with an effective 94.72% response rate. The study was conducted and reported in accordance with the Strengthening the Reporting of Observational Studies in Epidemiology (STROBE) guidelines.

### Study Instruments

The survey included three scales to collect the data about the study variables. The first part of the survey was a questionnaire that gathered data on nurses’ demographics (gender, age, and marital status) and professional characteristics (qualification, experience, and workshop (s) attendance). In the second part of the survey, a 10-item scale reported in the work of Yu et al. (2020) was used to measure OR nurses’ JE. The scale contributes to the three aspects of JE: link, fit, and sacrifice. Each item was rated on a 1–5 point Likert scale, with a higher score reflecting better JE. The composite reliability of the JE scales was 0.89 which indicates good internal consistency and reliability [42].

In the third part of the survey, the Polychronic-Monochronic Tendency Scale (PMTS) was used to evaluate polychronicity traits of the OR nurses. The PMTS was developed by Lindquist and Kaufman-Scarborough (2007), and consists of five items. Items were scored on a 5 point Likert scale, from one (strongly disagree) to five (strongly agree), with lower ratings indicating stronger monochronicity and higher scores indicating greater polychronicity. The Cronbach’s α values of the scale was 0.93 [46].

In the last part of the survey, the MISSCARE Survey-OR developed by Marsh et al. (2020) was used to assess OR nurses’ perceptions of missed care, either by themselves or by other staff members, during the preoperative or intraoperative phases of care. The survey is divided into two sections: Part A: MNC and Part B: Causes for MNC. Only Part A was used in this study. It consists of 32 items divided into five subscales: legal requirements, communication, preparation, safety, and closing routine. Internal reliability measured by Cronbach’s alpha of the five subscales ranged from 0.71 to 0.84. Each item is graded on a 5-point scale, with one being never missed and five always being missed [47].

### Tools adaption, validation and reliability

#### Tools translation

We have obtained the written permission from the original authors of the tools to use and translate them into Arabic. Tools were translated from English versions into Arabic versions using the back translation [48] to adapt to Egyptian culture, ensure accuracy and eliminate any potential threats to the study’s validity.

Following the translation of the tools, we employed various methods to evaluate their validity and reliability, such as content validity, exploratory factor analysis (EFA), confirmatory factor analysis (CFA), corrected item-total correlations, inter-dimension correlations, and Cronbach’s alpha. We used IBM SPSS software package version 22.0 and AMOS version 23.0 for the analyses (see supplementary).

#### Content validity

A committee of five academics from the field of study, comprising three professors in medical-surgical nursing and two professors in nursing administration, reviewed the tools for content validity. The expert panel pointed out punctuation and typing mistakes, and word choice issues. Some words were modified based on their recommendations. A pilot study was also conducted on 31 nurses to ensure the clarity and execution of the tools, as well as to establish the time required to complete the study questionnaire. The pilot sample was not included in the study sample.

#### Construct validity

the construct validity of the translated tools was tested using exploratory factor analysis (EFA) and the confirmatory factor analysis (CFA).

**Exploratory factor analysis** (EFA) using promax rotation with Kaiser Normalization was employed. The EFA aimed to identify the underlying factors or dimensions that explain the variance in the responses to the items of the each questionnaire.

The EFA of the MISSCARE Survey-OR revealed a clear and consistent factor structure that reflected the five dimensions of the scale with high boldface loadings for all items, ranging from 0.537 to 0.833 which means that the items strongly contributes to their factor. The Kaiser-Meyer-Olkin (KMO) measure of sampling adequacy was 0.918, which indicates the appropriateness of the data for factor analysis and that there is a high degree of common variance among the items.

The EFA of the PMTS using Kaiser Normalization revealed that there is one dominant factor that accounts for most of the variance in the responses. This factor has high loadings for all items, ranging from 0.689 to 0.777. This implies that it represents a general dimension of polychronic-monochronic tendency, and that all items measure the same construct. The KMO value is 0.824, indicates that the data are very suitable for factor analysis, and that there is a high degree of common variance among the items.

The EFA of the JE Scale revealed a clear and consistent factor structure that reflected the three sections of the tool, with high loadings for all items on the corresponding factor, ranging from 0.506 to 0.834. This means that the items are more clearly defined and distinct from each other, and that they measure different aspects of job embeddedness. The KMO value of 0.877 indicates that the data are very suitable for factor analysis, and that there is a high degree of common variance among the items.

**Confirmatory Factor Analysis**, CFA was done using structural equation modeling (SEM) for the mean and standard deviation of the MISSCARE Survey-OR and JE scale. The CFA aimed to test the fit of the factor structure derived from the EFA to the data. The CFA of MISSCARE Survey-OR confirmed that the model fit the data moderately well (comparative fit index (CFI) = 0.789, incremental fit index (IFI) = 0.790, root mean square error of approximation (RMSEA) = 0.080, Model *X*^2^ = 11.138^,^ p = .001*). The CFA of JE scale showed that the model fit the data very well (CFI = 0.952, IFI = 0.953, RMSEA = 0.063, Model X^2^ = 21.097^*^, P = .001*).

### Reliability

The reliability of the tools was assessed using the corrected item-total correlations, Inter-dimension correlations, and internal consistency.

**The corrected item-total correlations**, is the correlation between an item and the total score of the scale, excluding that item. It is a measure of how well each item fits with the overall scale. The corrected item-total correlations of the MISSCARE Survey-OR: showed that all items were positively and significantly related to their respective sections and to the overall score of the survey, ranging from 0.662 to 0.847. The corrected item-total correlations of the Polychronic-Monochronic Tendency Scale showed that all items were positively and significantly related to the scale, ranging from 0.666 to 0.780. The corrected item-total correlations of the JE Scale showed that all items were positively and significantly related to their respective sections and to the overall score of the tool, ranging from 0.699 to 0.820. This means that all items are positively related to their respective subscales, and that they measure the same construct as the other items in the subscale.

**Inter-dimension correlations**, measures correlations between the dimensions of each tool and the correlation between each dimension and the overall score, which is the sum of all items in the tool. For the MISSCARE Survey-OR, the results shows that all correlations are positive and statistically significant, indicating that there is a positive relationship between all dimensions and between each dimension and the overall score. However, some correlations are relatively low, such as between Part I and Part V (0.333), Part II and Part V (0.298), and Part II and Part IV (0.315). These low correlations suggest that some dimensions may not be very similar with each other, and that they may measure different aspects of missed nursing care. For the JE Scale, the results show that all correlations are positive and statistically significant, indicating that there is a positive relationship between all dimensions and between each dimension and the overall score. However, some correlations are relatively low, such as between Part I and Part II (0.560), Part II and Part III (0.479), and Part I and Part III (0.569). These low correlations suggest that some dimensions may not be very similar with each other, and that they may measure different aspects of JE.

**Internal consistency**, the Cronbach’s alpha was used to measure the internal consistency of the tools. It is a measure of how well a set of items measures a single construct or dimension. It ranges from 0 to 1, with higher values indicating higher reliability and internal consistency. The Cronbach’s alpha values for the subscales of the MISSCARE Survey-OR, ranged from 0.712 to 0.832 and 0.756 for the overall survey. The Cronbach’s alpha of PMTS was 0.778. The Cronbach’s alpha values for all subscales of JE scale ranged from 0.728 to 0.841and the overall scale was 0.834. These results indicate acceptable reliability and internal consistency of the three study tools.

### Data collection

Before data collection the Research Ethics Committee of Faculty of Nursing, Alexandria University and the responsible authorities of the selected setting, approved the study protocol. The researchers collected data by hand-delivering anonymous questionnaires to participants after obtaining informed consent to participate. The questionnaire took an average of 30 min to be completed. We collected the data on May and June 2023.

### Statistical analysis of the data

Data were analyzed using IBM SPSS 22.0 and PROCESS macro version 4.2 for SPSS. We screened the data for respondent misconduct (e.g. extreme or multiple responses) and the screening was good and all responses were considered for analysis. For descriptive statistics, mean and standard deviation were used to present continuous variables, while categorical variables are presented by frequency and percent. The Pearson coefficient was used to correlate normally distributed quantitative variables. A path analysis was carried out using PROCESS macro- model 4 to examine whether the effect of JE (X) on missed care at operating theatres (Y) was mediated by polychronicity (M). Initially, we controlled for participant characteristics due to their probable relationship with the dependent variable. The analysis revealed a nonsignificant association; thus, the model was estimated using the main study variables. Bootstrapping (5000 samples) with a 95% confidence interval was used to determine the statistical significance of the indirect mediation effect on missed care. The indirect effect was considered statistically significant if the 95% confidence interval (CI) did not include zero.

## Results

### Demographics and professional characteristics of the study participants

The data presented in Table 1 show that the majority of the respondents were female (62.2%) and aged between 20 and 30 years (46.6%). The mean age was 33.56 years, with a standard deviation of 9.97. In terms of years of experience in nursing, the majority of respondents had less than 5 years of experience (40.5%), with a mean of 12.01 years and a standard deviation of 10.86. For years of experience in operating theaters, the majority had less than 5 years of experience (44.3%), with a mean of 9.76 years and a standard deviation of 9.37. In terms of qualification, the majority had a bachelor’s degree (47.2%), followed by a diploma (36.7%). The majority of respondents were married (53.7%), followed by single (33.1%). Finally, more than half of the respondents reported attending previous workshops regarding perioperative care (50.7%).

**Table 1:** Distribution of the studied sample according to demographic and professional characteristics (n = 341)

	No	%
**Age**		
20–30	159	46.6
> 30–40	82	24.0
> 40–50	72	21.1
> 50	28	8.2
**Mean ± SD**	**33.56 ± 9.97**	
**Gender**		
Male	129	37.8
Female	212	62.2
**Years of experience in nursing**		
< 5	138	40.5
5-<10	62	18.2
10-<20	44	12.9
≥ 20	97	28.4
**Mean ± SD**	**12.01 ± 10.86**	
**Years of experience in operating theatres**		
< 5	168	44.3
5-<10	61	17.9
10-<20	41	12.0
≥ 20	71	20.8
**Mean ± SD**	**9.76 ± 9.37**	
**Qualification**		
Diploma	125	36.7
Bachelor	161	47.2
Specialized diploma	30	8.8
Masters	17	5.0
PhD	8	2.3
**Marital status**		
Single	113	33.1
Married	183	53.7
Widowed	25	7.3
Divorced	20	5.9
**Attending workshop**		
Yes	173	50.7
No	168	49.3

### Descriptive statistics and correlations of the study variables

The data presented in Table 2 show the descriptive statistics of the MISSCARE Survey-OR. The average of the survey composite score was 2.96 ± 0.35, as the highest mean score was for communication (3.19 ± 0.54), while the lowest mean score was for closing routines (2.43 ± 0.60). In great detail, OR nurses rated the surgical time out occurred in the OR with the participation of all members of surgical team (3.53 ± 0.1.05), the nurse documented patient’ allergies and reported it to the surgical team (3.5 ± 1.03), a standardized communication tool was utilized for handovers (3.48 ± 1.17), application of sequential compression devices were done before the beginning of surgery (3.37 ± 0.99), the nurse used two unique identifiers to confirm the patient’s identity (3.3 ± 0.85), and thorough handover occurred between team members before transferring the patient to the OR (3.31 ± 1.07), as the most frequently missed care. On the other hand, count discrepancies being shared with the surgeon (2.18 ± 0.8), specimens being sent to the laboratory (2.32 ± 0.98), required blood products being ordered and prepared before surgery (2.36 ± 0.95), and when monopolar electrosurgery is employed, a single-use dispersive electrode being used (2.36 ± 1.11) were rated as less likely missed care at the operating theaters.

The data provided in Table 3 show that the average composite score of the PMTS was 2.79 ± 0.75. Table 4 reveals that the average nurse’s JE scale was 2.92 ± 0.57, with the greatest subscale scores for link (3.4 ± 0.67) and the lowest for fit (2.92 ± 0.57).

**Table 2:** Mean and Standard Deviation for MISSCARE Survey-OR, (n = 341)

MISSCARE Survey-OR	Mean ± SD
**Legal requirements**	3.08 ± 0.56
1. The patient’s identity is confirmed by the nurse using at least two unique identifiers	3.30 **±** 0.85
2. The patient or patient’s legal custodian is requested by the nurse to confirm the surgical procedure	3.11 **±** 0.93
3. The nurse ensures that the surgical consent is signed, dated, and witnessed before surgery	2.76 **±** 0.96
4. The nurse confirms with the patient or patient’s legal custodian that the right procedure is on the consent	3.04 **±** 0.98
5. Marking the correct surgical site by surgeon is verified by the nurse	3.12 **±** 1.03
6. The nurse confirms that history and physical examinations is fulfilled and dated before surgery	3.13 **±** 1.00
**Preparation**	**2.99 ± 0.44**
7. The nurse document patient’ allergies and reported it to the surgical team	3.50 **±** 1.03
8. The nurse reports abnormal laboratory findings to the anesthesiologists	2.88 **±** 1.12
9. The nurse reports patient’ latex allergy to surgical team	2.98 **±** 1.01
10. The patient’s and legal custodian’s inquiries regarding surgery are met by the surgeon and the nurse	3.08 **±** 1.04
11. The patient is provided by comfort measures	3.26 **±** 0.97
12. Implantable devices are identified and reported to the surgical team	2.62 **±** 0.91
13. Patients with infectious diseases are isolated using universal isolation precautions	3.00 **±** 1.04
14. Blood and blood components required are prepared before surgery	2.36 **±** 0.95
15. Prophylactic antibiotics are prescribed and administered to the patient before surgery if indicated	2.56 **±** 0.78
16. Application of sequential compression devices is done before the beginning of surgery, if applicable	3.37 **±** 0.99
17. Complete handover occurs among team members before transferring the patient to the OR	3.31 **±** 1.07
**Safety**	**2.74 ± 0.43**
18. Transferring patient to and from the OR bed occurs without injury	2.61 **±** 0.98
19. Positioning of patient is done in a manner that prevent potential complications	2.79 **±** 1.01
20. Surgical team confirms patient, consent, procedure, site, and side before incision	3.11 **±** 1.02
21. When the monopolar electro surgery is used, a single-use dispersive electrode is applied	3.36 **±** 1.11
22. Monitoring the surgical field for breaks in aseptic technique is done	2.39 **±** 0.77
23. Count differences are notified to the surgical team	2.18 **±** 0.8
**Communication**	**3.19 ± 0.54**
24. All activities are stopped during the surgical time out	3.15 **±** 1.01
25. Surgical time out occurs in the OR with the participation of all members of surgical team	3.53 **±** 1.05
26. Team members’ quires or concerns are addressed before the incision is made	2.95 **±** 1.13
27. The team waits three minutes for dryness of alcohol-based antiseptic solutions used for skin preparation	3.06 **±** 1.20
28. Hand overs occurs using standardized endorsement tool	3.48 **±** 1.17
29. Essential information is handed over among surgical professionals at the times of breaks, lunch, shift changes	2.97 **±** 1.18
**Closing routine**	**2.43 ± 0.60**
30. Surgical counts are confirmed as correct during closing	2.47 **±** 1.03
31. The nurse verifies and labels all specimens correctly	2.50 **±** 1.08
32. The nurse send specimens to the laboratory	2.32 **±** 0.98
**MISSCARE Survey-OR**	**2.96 ± 035**

**Table 3:** Mean and Standard Deviation for Polychronic- Monochronic Tendency Scale, (n = 341)

Polychronic-Monochronic Tendency Scale	Mean ± SD
1. I prefer to do two or more activities at the same time.	2.83 ± 1.02
2. I typically do two or more activities at the same time.	2.43 ± 0.84
3. Doing two or more activities at the same time is the most efficient way to use my time.	2.83 ± 1.10
4. I am comfortable doing more than one activity at the same time.	2.94 ± 1.10
5. I like to juggle two or more activities at the same time.	2.94 ± 1.06
**Polychronic -Monochronic Tendency Scale**	**2.79 ± 0.75**

**Table 4:** Mean and standard deviation for Job Embeddedness Scale, (n = 341)

Job Embeddedness scale	Mean ± SD
**Fit**	2.67 ± 0.68
1. My job utilizes my skills and talents well.	2.67 ± 0.90
2. I fell I am a good match for this organization.	2.64 ± 0.87
3. My organization highly evaluates my ability.	2.70 ± 0.89
4. I fit with this organization’s working style.	2.65 ± 0.92
**Links**	**3.40 ± 0.67**
5. I have a good relationship with other employees in this organization	3.46 ± 0.75
6. I have a good communication with employees in other teams.	3.49 ± 0.84
7. I am a member of many social teams in this organization.	3.24 ± 0.89
**Sacrifice**	**2.79 ± 0.69**
8. This organization provides lots of well-being benefits to employees.	2.77 ± 0.95
9. I am well compensated for my level of performance.	2.80 ± 0.89
10. I will sacrifice many things if I leave this organization.	2.79 ± 1.02
**Job Embeddedness**	**2.92 ± 0.57**

Table 5 shows the association between the studied variables. There was a moderate, negative, and significant association between the missed care at the operating theaters measured by the MISSCARE survey-OR and the polychronic-monochronic tendency of the OR nurses (*r* = -.687, *p* < .001), with a significant association between the polychronic-monochronic tendency and all subscales of the MISSCARE-OR survey (*r*-value ranged from − 0.886 to -0.197, *p* < .001). Similarly, a moderate, negative, and significant association was observed between JE and overall missed care at the OR (*r* = -.428, *p* < .001). There was a negative and significant association between overall JE and all subscales of missed care at the OR (*r*-value ranged from − 0.428 to -0.144, *p* < .001). In addition, there was a negative and significant correlation between the overall missed care at the OR and all subscales of JE, which were fit (*r* = -.315, *p* < .001), links (*r* = -.227, *p* < .001), and sacrifice (*r* = -.539, *p* < .001).

Furthermore, there was a moderately positive and significant association found not only between the polychronic-monochronic tendency of the OR nurses and their overall JE (*r* = .522, *p* < .001) but also between the polychronic-monochronic tendency and all subscales of JE, which were fit (*r* = .319, *p* < .001), links (*r* = .305, *p* < .001), and sacrifice (*r* = .717, *p* < .001) (see Table 5).

**Table 5:** Correlation between the Study Variables (N = 341)

		1	2	3	4	5	6	7	8	9	10
1. **Legal requirements**	r										
	*p*										
3. **Preparation**	r	0.609*									
	*p*	< 0.001									
5. **Safety**	r	0.308*	0.382*								
	*p*	< 0.001	< 0.001								
7. **Communication**	r	0.402*	0.458*	0.337*							
	*p*	< 0.001	< 0.001	< 0.001							
9. **Closing routine**	r	0.046	0.088	0.135*	0.110*						
	*p*	0.392	0.105	0.013	0.042						
11. **MISSCARE Survey-OR**	r	0.753*	0.843*	0.609*	0.732*	0.279*					
	*p*	< 0.001	< 0.001	< 0.001	< 0.001	< 0.001					
13. **Polychronic -Monochronic tendency**	r	− 0.531*	− 0.586*	− 0.402-*	− 0.501*	− 0.197*	− 0.687*				
	*p*	< 0.001	< 0.001	< 0.001	< 0.001	< 0.001	< 0.001				
15. **Fit**	r	− 0.203*	− 0.275*	− 0.232-*	− 0.232*	− 0.096	− 0.315*	0.319*			
	*p*	< 0.001	< 0.001	< 0.001	< 0.001	0.076	< 0.001	< 0.001			
17. **Links**	r	− 0.177*	− 0.201*	− 0.122-*	− 0.168*	− 0.038	− 0.227*	0.305*	0.560*		
	*p*	0.001	< 0.001	0.025	0.002	0.481	< 0.001	< 0.001	< 0.001		
19. **Sacrifice**	r	− 0.397*	− 0.445*	− 0.341-*	− 0.378*	− 0.231*	− 0.539*	0.717*	0.569*	0.479*	
	*p*	< 0.001	< 0.001	< 0.001	< 0.001	< 0.001	< 0.001	< 0.001	< 0.001	< 0.001	
21. **Job Embeddedness**	r	− 0.305*	− 0.365*	− 0.279-*	− 0.309*	− 0.144*	− 0.428*	0.522*	0.885*	0.796*	0.807*
	*p*	< 0.001	< 0.001	< 0.001	< 0.001	0.008	< 0.001	< 0.001	< 0.001	< 0.001	< 0.001

**r**: Pearson coefficient *: Statistically significant at *p* ≤ .05.

### Mediating role of polychronicity on the relationship between job embeddedness and missed care at the operating theatres

**Fig. 2:**
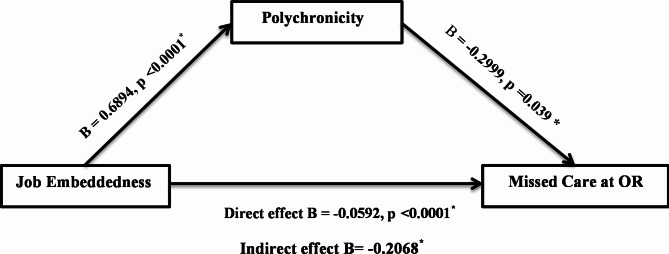
Path Estimates of the Direct and Indirect Effect of Job Embeddedness on Missed Care at the Operating Theatres Mediated by Role of Polychronicity (n = 341)

As shown in Table Table 6; Fig. 2, the model path results showed that nurses’ JE had a positive effect on the polychronic tendency of OR nurses (B = 0.6894, *p* < .0001), supporting H1. JE explained 27.3% of the variance in their polychronic tendency. JE had a direct negative effect on the perceived missed care at the operating theaters (B = -0.0592, *p* < .0001), supporting H2. Polychronic tendency negatively influenced their perceived missed care (β = -0.4786, *p* = .039), supporting H3. Thus, JE and polychronicity were predictors of perceived missed care at the OR. Together, they explained 47.86% of the variance in perceived missed care at the operating theaters.in addition the table shows that the total effect of OR nurses’ JE on their perceived missed care at the operating theaters was significant (β = -0.266, *p* < .0001). There was a significant indirect impact of JE on perceived missed care mediated by the role of polychronicity (B = -0.2068, 95% CI = -0.255 to -0.163), accounting for 77.74% of the total effect with these results supporting H4. Hence, polychronicity partially mediated the relationship between JE and missed care at the operating theaters.

**Table 6:** Mediating role of Polychronicity in the Relation between Job Embeddedness and Missed Care, (n = 341)

Dependent variables		Independent variables	B	R-sq	S.E	95% Confidence interval		t	p value	Effect proportion
						LL	UL			
**Model path**	**Polychronicity**	**Job Embeddedness**	0.6894	0.2730	0.061	0.569	0.809	11.28	< 0.0001^*^	-
	**Missed care**	**Polychronicity**	-0.2999	0.4786	0.021	-0.342	-0.257	-13.83	0.039 ^*^	-
	**Missed care**	**Job Embeddedness**	-0.0592		0.028	-0.115	-0.003	-2.07	< 0.0001^*^	22.26%
**Type of effect**	**Direct effect**									
	**Indirect effect**		-0.2068		0.0236	-0.255	-0.163	-	(sig)	77.74%
	**Total effect**		-0.2660		− 0.0305	-0.326	-0.206	-8.726	< 0.0001^*^	-

**B**: Unstandardized regression coefficient; **SE**: standard error, **LL**: lower limit, **UL**: upper limit *: statistically significant at *p* ≤ .05, 5000 bootstrap samples used

## Discussion

Perioperative nursing care is a significant part of nursing practice that may be affected by numerous factors leading to the missing of necessary care that further impede patient safety. The present study aims to investigate the mediating role of polychronicity on the relationship between JE and MNC at operating theaters. The study assumes that polychronicity, which is the ability to perform multiple tasks simultaneously, may be a mediator between JE and MNC. The study is based on the assumption that nurses with a high level of JE tend to be more committed to their job, which may reduce MNC. However, the study hypothesizes that this relationship might be affected by polychronicity, which allows nurses to juggle multiple tasks and, therefore, be more efficient.

Our study revealed that many different and important aspects of perioperative nursing care were missed. Communication, legal requirements, and preparation are the highest areas where nurses and surgical teams missed vital elements. Surgical time out, reporting and documentation of allergies, and standardized handover are the most frequent items that were missed among nurses at OR. This could be attributed to staffing shortage especially among nurses, the high workload in operating theatres where there are numerous scheduled operations in the daily list of the study settings and starting more than seven operations at one time.

One possible explanation of these items of missed perioperative nursing care is the negative attitudes of staff toward using safe surgical checklist which is considered a metaphor for steps for safer surgery. This negative attitude was highlighted in studies conducted in the study settings [49–51]. Poor prevailing patient safety culture is a another concern explaining missed perioperative nursing care since previous works in the study settings found poor perception of healthcare providers for patient safety culture [52–54]. Poor staff compliance with patient safety guidelines as well as lack of awareness about risk mitigation are also critical factors explaining the missed care items in our study. These factors are prominent findings in studies conducted in the study settings [55, 56].

The complexity of operations conducted in study settings, time pressure associated with the surgical team’s fear of causing a cascading delay in procedure start times, and scarcity of resources are other possible reasons behind MNC in communication and preparatory phases of perioperative nursing care. These reasons were also reported in other studies [33, 34, 47, 57]. In addition, Diab and Ebrahim (2019) and Zeleníková et al. (2019) found that a shortage of labor resources is the greatest factor contributing to MNC [58, 59].

On the other hand, closing routines and safety are the areas with the least missed care. Specifically, reporting count discrepancies to the surgeon, labeling, and handling of specimens are the least missed perioperative items. This may be due to the increased focus of Egyptian state on patient safety issues and preventing surgical errors. Additionally, ending the surgical procedure is associated with low levels of stress, which in turn helps the surgical team to finish necessary items completely and properly. Our findings are consistent with the study of Beitz, (2019), who found that nurses often missed items about the communication and preparation of patients before surgical procedures [58]. In addition, in the study conducted on 1693 nurses working in operating theatres, nurses reported the most missed care in the communication and preparation categories, whereas up to 2.5% of nurses reported that the least missed items were those related to safety in surgical theatres, such as the transfer of the patient to and from the OR bed without harm and communication of surgical count discrepancies to the surgeon [47].

In this context, an investigation of missed perioperative nursing care in 17 Michigan hospitals showed that communication failure among the surgical team is the most significant part, followed by patient identification and marking the site of surgery. In addition, aspects of missed care varied according to the type surgery. Gallbladder surgeries had the most reported missed care items, whereas breast cancer surgeries had the fewest [60]. Additionally, a systemic review conducted on 5,134 nurses working at 138 units in 14 US hospitals assessed the extent and type of MNC and reasons for the missed care and found that items related to patient safety in operating theatres were the least missed, whereas preparation and communications items, especially reporting abnormal laboratory values immediately before surgery to anesthesia professionals, identification of implantable devices, and hand over communication between operating theatres and other departments, were the prominent missed items [61].

MNC in operating theatres is a critical variable that is affected by the personal and professional factors of OR nurses, such as their JE and their polychronicity. Our results revealed that there is a moderate, negative, and significant association between the JE of OR nurses, polychronicity, and missed perioperative nursing care. Moreover, JE and polychronicity are powerful predictors of missed perioperative nursing care since they account for 47.8% of the variance in missed perioperative nursing care. This means that OR nurses who are embedded in their jobs and have a high polychronic tendency are less likely to forget perioperative nursing care. In other words, missed perioperative nursing care is less likely to occur among OR nurses who are embedded in their practice and exhibit a high propensity to engage in multiple tasks simultaneously. This relationship is expected since earlier studies found that embedding nurses in their jobs is associated with many positive results because they have to address varying patient needs [28, 62]. Moreover, polychronic nurses show high performance in different contexts in which there are no failures or omissions of critical elements of nursing care [63].

This relationship could be explained and supported by the assumptions of reasoned action theory and congruence model discussed earlier. In addition, Kim and Park (2023) found embedded nurses in their jobs often seek perfectionism and demonstrate multiple competencies this also could provide a reasonable explanation why JE could reduce missed perioperative nursing care [64]. Meanwhile, Pan et al. (2023) found employees who act polychronically able to control work interruptions successfully and have high performance scores than those who act monochronically. Furthermore, they able to make robust prioritization in their daily activities which could explain why polychronicity is associated with less missed perioperative nursing care [65].

Our findings are in line with different studies that shed light on the relationship between JE and several aspects of nursing care, including missed perioperative care. For example, Albsoul et al. (2019) found that nursing staff satisfaction, JE, and engagement were significant factors in shaping the extent to which nursing care was missed [66]. The studies of Lake et al. (2020) and Hessels et al. (2015) revealed that a positive work environment in which nursing staff are embedded with their jobs and their organizations was associated with lower levels of MNC [67, 68]. Additionally, the study of Elpasiony and Abd-Elmoghith (2023) found that higher levels of JE among nurses were associated with better quality of care and a lower risk of patient safety incidents [40]. Similarly, a study by Kang et al. (2018) found that higher levels of JE among nurses were associated with fewer medication errors [69].

Some studies have also investigated the effect of polychronicity on nursing care and patient safety, and their results agree with our findings. A study by Kim et al. (2018) found that nurses who were able to perform multiple tasks simultaneously were less likely to experience burnout, which is a predictor of MNC [57]. Another study by Pachler et al. (2018) found that nurses who had high levels of polychronicity were more effective in managing interruptions during medication administration [70]. Furthermore, a scoping review of 34 studies revealed that nurse characteristics such as the polychronic tendency are associated with low levels of omitted care in all contexts [71]. However, some studies have also suggested that polychronicity may have a negative effect on nursing care. For example, a study by Rottapel (2017) found that nurses who multitasked frequently reported more MNC and lower job satisfaction [72]. Another study by Berg (2018) found that nurses who multitasked frequently were more likely to make medication errors [73].

Concerning the role of polychronicity in the relationship between nurses’ JE and MNC in operating theatres, our study found that polychronicity partially mediated this relationship. In other words, the JE of OR nurses plays a significant role in reducing missed perioperative nursing care mediated by the polychronic tendency of nurses (77.7% of the total effect). This means that polychronicity acts as a powerful mediator in the effect of JE on missed perioperative care among OR nurses.

This mediating role of polychronicity is supported by the positive significant relationship yielded in our study between the polychronic tendency among OR nurses and their JE, which reflects that nurses who are embedded in their jobs can perform multitasks simultaneously in an efficient manner. Efficiency in this case is a guarantee that basic and critical elements of nursing care are not missed. Our findings give an impression of the importance of strategies that could make nurses attached and embedded in their jobs as well as foster the polychronic tendency among nurses. These strategies could significantly improve the quality of nursing care and reduce the margins of MNC at operating theatres.

It is important to note that results of the mediating role of polychronicity in our study is considered as an extension to and is supported by the reasoned action theory. Accordingly, when the nurse embedded in his/ her job, she /he could act polychronically. Acting polychronically means that the nurse able to consider minor and major details of tasks assigned to him/her simultaneously. This further explain why polychronicity could reduce incidence of missed perioperative nursing care and also could justify the powerful mediating role of polychronicity on the effect of JE on missed perioperative nursing care.

Our results are similar to the findings of Asghar et al. (2020) and Yousaf et al. (2021, these studies found that the polychronic behavior of nurses was associated with innovative behavior, which in turn helped to reduce missed perioperative care [63, 74]. Pachler et al. (2018) highlighted that polychronic nurses are always struggling search for novel ideas to improve the quality of their practice in which omissions are not expected [70]. Waheed et al.(2021) found that polychronic nurses were creative, engaged in their work and had a high level of work performance [75]. Additionally, the study conducted in Iran revealed that the disengagement of nurses from their jobs was associated with increased missed critical pillars of nursing care [76]. Furthermore, a Chinese study concluded that making employees embedded in their jobs could enable them to master multitasks. The high polychronic tendency among engaged employees is associated with high conduct; hence, there is no room for missing tasks [77].

Overall, MNC at operating theatres is inevitable due to the complexity of its antecedents. However, selecting nurses with high polychronic tendency as well as creating a practice environment that can make nurses embedded and engaged with their roles are golden strategies to reduce the magnitude of missed perioperative nursing care.

### Implications of the study

Our study offers new insights for nursing management and healthcare practitioners to reduce the incidence of MNC at operating theatres. The study suggests that cultivating an environment where nurses feel attached to their organization, job, and colleagues can reduce MNC in ORs. Additionally, recruiting and selecting an adequate number of OR nurses who have a high polychronic tendency is a valuable approach to mitigate future missed perioperative nursing care. Moreover, nurses should be provided with the opportunity and training to develop their polychronic skills to manage multiple tasks effectively and efficiently. Furthermore, nurses should be provided with the necessary support and resources required to engage in polychronic behavior.

By acknowledging the potential for oversight, implementing effective communication strategies, and utilizing standardized protocols, we can work toward minimizing missed care occurrences in operating theaters. Striving for comprehensive and patient-centered care throughout the perioperative period will undoubtedly contribute to enhanced patient care outcomes and safeguarding patient safety. In addition, utilizing time management tools, prioritizing necessary tasks, conducting inventories, appropriately distributing workload, and controlling interruptions and distractions are vital considerations that should be taken by surgical teams to ensure that critical elements of perioperative care are not missed.

Our study has several implications for theory and nursing practice. It contributes to the literature by providing research evidence about novel strategies to reduce MNC in the OR context. Additionally, it extends the literature on polychronic behavior by examining its interplayed effect along with JE in reducing MNC at operating theatres. Previous studies address these variables separately or through bivariate analysis only. Finally, it highlights the importance of enhancing polychronic behavior among nurses as a way to ultimately improve the quality of nursing care.

### Study strengths and limitations

Our study offers new insights on how to minimize the occurrence of missed nursing care at operating theatres. It is the first study to explore the factors that influence missed nursing care at operating theatres, such as job embeddedness and polychronicity. It contributes to the nursing literature by empirically testing the effects of these variables on MNC. It also suggests practical strategies and actions for nurse leaders and clinicians to enhance safety and quality in operating theatres. Moreover, our study aligns with the sustainable development goals by emphasizing both safety and human welfare. Our study has high reliability as it was conducted in multiple sites of operating theatres and used robust statistical methods to ensure the validity and reliability of the instruments used.

This study also has some limitations that should be considered. First, the study employed a cross-sectional design, which limits the ability to demonstrate causal relationships among the variables. Future studies should use longitudinal or experimental designs to test the causal effects of polychronicity and JE on MNC and patient care outcomes. Second, data collected from nurses used self-report measures that may introduce common method bias or social desirability bias. Third, the study used 341 nurses working in ORs, which is a limited sample size although we recruited all target populations in the study settings. This may limit the generalizability of the findings.

## Conclusion

Our study tested the impact of polychronicity on the intermediate linkage between OR nurses’ JE and missed perioperative nursing care. It is concluded that polychronicity is a powerful mediator between JE and MNC in the ORs. In addition, polychronic nurses are more embedded in their jobs. When OR nurses’ traits such as polychronicity interplayed with JE, a significant decrease in the amount of missed perioperative nursing care is a possible outcome.

### Electronic supplementary material

Below is the link to the electronic supplementary material.

Supplementary Material 1

## Data Availability

The datasets used and/or analysed during the current study are available from the corresponding author on reasonable request.

## List of abbreviations

**OR**: Operating room
**JE**: Job embeddedness
**MNC**: Missed nursing care
**PMTS**: Polychronic-Monochronic Tendency Scale
