# Supplementary material for: Job embeddedness and missed nursing care at the operating theatres: the mediating role of polychronicity
Source: BMC Nurs. 2023 Dec 4;22:458. doi: 10.1186/s12912-023-01628-8 (PMC10696744; doi:10.1186/s12912-023-01628-8)
Supplement: Supplementary file 1 — Supplementary Material 1 [file 12912_2023_1628_MOESM1_ESM.docx]

**Table 1s: Exploratory factor analysis (EFA) and factor loadings of MISSCARE Survey-OR**

|  |  | **After rotation Promax with Kaiser Normalization** | | | | | **Communalities** |
| --- | --- | --- | --- | --- | --- | --- | --- |
| **Item number** | **Section** | **Factor 1** | **Factor 2** | **Factor 3** | **Factor 4** | **Factor 5** |  |
| item1 | Part I |  | 0.656 |  |  |  | 0.541 |
| item2 | Part I |  | 0.734 |  |  |  | 0.586 |
| item3 | Part I |  | 0.798 |  |  |  | 0.654 |
| item4 | Part I |  | 0.833 |  |  |  | 0.713 |
| item5 | Part I |  | 0.731 |  |  |  | 0.557 |
| item6 | Part I |  | 0.758 |  |  |  | 0.593 |
| item7 | Part 2 | 0.676 |  |  |  |  | 0.547 |
| item8 | Part 2 | 0.733 |  |  |  |  | 0.592 |
| item9 | Part 2 | 0.716 |  |  |  |  | 0.522 |
| item10 | Part 2 | 0.764 |  |  |  |  | 0.617 |
| item11 | Part 2 | 0.705 |  |  |  |  | 0.516 |
| item12 | Part 2 | 0.710 |  |  |  |  | 0.582 |
| item13 | Part 2 | 0.685 |  |  |  |  | 0.525 |
| item14 | Part 2 | 0.642 |  |  |  |  | 0.648 |
| item15 | Part 2 | 0.712 |  |  |  |  | 0.591 |
| item16 | Part 2 | 0.701 |  |  |  |  | 0.517 |
| item17 | Part 2 | 0.696 |  |  |  |  | 0.548 |
| item18 | Part 3 |  |  |  | 0.805 |  | 0.658 |
| item19 | Part 3 |  |  |  | 0.718 |  | 0.567 |
| item20 | Part 3 |  |  |  | 0.665 |  | 0.508 |
| item21 | Part 3 |  |  |  | 0.481 |  | 0.378 |
| item22 | Part 3 |  |  |  | 0.760 |  | 0.673 |
| item23 | Part 3 |  |  |  | 0.792 |  | 0.647 |
| item24 | Part 4 |  |  | 0.609 |  |  | 0.486 |
| item25 | Part 4 |  |  | 0.719 |  |  | 0.543 |
| item26 | Part 4 |  |  | 0.537 |  |  | 0.396 |
| item27 | Part 4 |  |  | 0.650 |  |  | 0.449 |
| item28 | Part 4 |  |  | 0.642 |  |  | 0.434 |
| item29 | Part 4 |  |  | 0.715 |  |  | 0.556 |
| item30 | Part 5 |  |  |  |  | 0.777 | 0.633 |
| item31 | Part 5 |  |  |  |  | 0.822 | 0.683 |
| item32 | Part 5 |  |  |  |  | 0.830 | 0.703 |
| **Kaiser-Meyer-Olkin Measure of Sampling Adequacy = 0.918** | | | | | | | |

The boldface indicates salient (> 0.30) loading


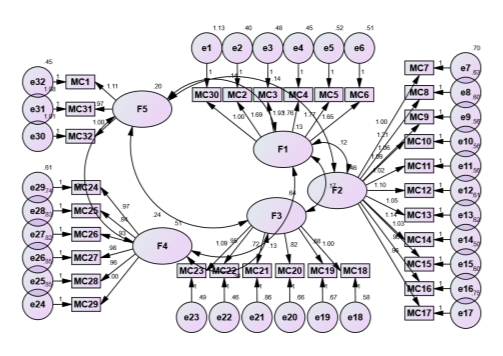


**Figure 1s: Confirmatory factor analysis (CFA) by Structure Equation Modeling for Mean and Standard Deviation for MISSCARE Survey-OR**

Model fit parameters CFI; IFI; RMSEA (0.789; 0.790; 0.080).

CFI = Comparative fit index; IFI = incremental fit index; and RMSEA = Root Mean Square Error of Approximation.

Model ^^; significance 11.138^*^(0.001*)

**Table 2s: Corrected Item-Total Correlations for MISSCARE Survey-OR**

| **Item number** | **Section** | **Correlation** |
| --- | --- | --- |
| item1 | Part I | 0.720^*^ |
| item2 | Part I | 0.781^*^ |
| item3 | Part I | 0.795^*^ |
| item4 | Part I | 0.800^*^ |
| item5 | Part I | 0.766^*^ |
| item6 | Part I | 0.767^*^ |
| item7 | Part 2 | 0.675^*^ |
| item8 | Part 2 | 0.751^*^ |
| item9 | Part 2 | 0.713^*^ |
| item10 | Part 2 | 0.737^*^ |
| item11 | Part 2 | 0.708^*^ |
| item12 | Part 2 | 0.735^*^ |
| item13 | Part 2 | 0.707^*^ |
| item14 | Part 2 | 0.720^*^ |
| item15 | Part 2 | 0.732^*^ |
| item16 | Part 2 | 0.693^*^ |
| item17 | Part 2 | 0.662^*^ |
| item18 | Part 3 | 0.780^*^ |
| item19 | Part 3 | 0.725^*^ |
| item20 | Part 3 | 0.707^*^ |
| item21 | Part 3 | 0.635^*^ |
| item22 | Part 3 | 0.779^*^ |
| item23 | Part 3 | 0.800^*^ |
| item24 | Part 4 | 0.716^*^ |
| item25 | Part 4 | 0.660^*^ |
| item26 | Part 4 | 0.669^*^ |
| item27 | Part 4 | 0.687^*^ |
| item28 | Part 4 | 0.684^*^ |
| item29 | Part 4 | 0.704^*^ |
| item30 | Part 5 | 0.831^*^ |
| item31 | Part 5 | 0.844^*^ |
| item32 | Part 5 | 0.847^*^ |

Correlation: Pearson coefficient *: Statistically significant at p ≤ 0.05

**Table 3s: Correlations Between MISSCARE Survey-OR dimensions**

|  | | **Part 1** | **Part 2** | **Part 3** | **Part 4** | **Part 5** | **Overall MISSCARE Survey-OR** |
| --- | --- | --- | --- | --- | --- | --- | --- |
| **Part 1** | **r** |  |  |  |  |  |  |
|  | **p** |  |  |  |  |  |  |
| **Part 2** | **r** | 0.646^*^ |  |  |  |  |  |
|  | **p** | <0.001^*^ |  |  |  |  |  |
| **Part 3** | **r** | 0.668^*^ | 0.592^*^ |  |  |  |  |
|  | **p** | <0.001^*^ | <0.001^*^ |  |  |  |  |
| **Part 4** | **r** | 0.465^*^ | 0.315^*^ | 0.553^*^ |  |  |  |
|  | **p** | <0.001^*^ | <0.001^*^ | <0.001^*^ |  |  |  |
| **Part 5** | **r** | 0.333^*^ | 0.298^*^ | 0.400^*^ | 0.465^*^ |  |  |
|  | **p** | <0.001^*^ | <0.001^*^ | <0.001^*^ | <0.001^*^ |  |  |
| **Overall MISSCARE Survey-OR** | **r** | 0.803^*^ | 0.734^*^ | 0.837^*^ | 0.734^*^ | 0.692^*^ |  |
|  | **p** | <0.001^*^ | <0.001^*^ | <0.001^*^ | <0.001^*^ | <0.001^*^ |  |

r: Pearson coefficient *: Statistically significant at p ≤ 0.05

**Table 4s: Cronbach's Alpha for MISSCARE Survey-OR and its Subscales**

|  | **N of Items** | **Cronbach’s alpha** |
| --- | --- | --- |
| Part 1 | 6 | 0.723 |
| Part 2 | 11 | 0.821 |
| Part 3 | 6 | 0.832 |
| Part 4 | 6 | 0.735 |
| Part 5 | 3 | 0.712 |
| **MISSCARE Survey-OR** | **32** | **0.756** |

**Table 5s: Exploratory factor analysis (EFA) and factor loadings of Polychronic-Monochronic Tendency Scale**

|  | **Kaiser Normalization** | **Communalities** |
| --- | --- | --- |
| **Item number** | **Factor 1** |  |
| item1 | 0.698 | 0.487 |
| item2 | 0.689 | 0.475 |
| item3 | 0.770 | 0.593 |
| item4 | 0.777 | 0.604 |
| item5 | 0.706 | 0.498 |
| **Kaiser-Meyer-Olkin Measure of Sampling Adequacy = 0.824** | | |

The boldface indicates salient (> 0.30) loading

**Table 6s: Corrected Item-Total Correlations for Polychronic-Monochronic Tendency Scale**

| **Item number** | **Correlation** |
| --- | --- |
| item1 | 0.703^*^ |
| item2 | 0.666^*^ |
| item3 | 0.771^*^ |
| item4 | 0.780^*^ |
| item5 | 0.717^*^ |

Correlation: Pearson coefficient *: Statistically significant at p ≤ 0.05

**Table 7s: Cronbach's Alpha for Polychronic-Monochronic Tendency Scale**

|  | **N of Items** | **Cronbach’s alpha** |
| --- | --- | --- |
| **Polychronic-Monochronic Tendency Scale** | **5** | **0.778** |

**Table 8s: Exploratory factor analysis (EFA) and factor loadings of Job Embeddedness scale**

|  |  | **After rotation Promax with Kaiser Normalization** | | | |
| --- | --- | --- | --- | --- | --- |
| **Item number** | **Section** | **Factor 1** | **Factor 2** | **Factor 3** | **Communalities** |
| item1 | Part I | 0.674 |  |  | 0.504 |
| item2 | Part I | 0.831 |  |  | 0.729 |
| item3 | Part I | 0.729 |  |  | 0.595 |
| item4 | Part I | 0.740 |  |  | 0.568 |
| item5 | Part 2 |  | 0.825 |  | 0.713 |
| item6 | Part 2 |  | 0.812 |  | 0.670 |
| item7 | Part 2 |  | 0.742 |  | 0.627 |
| item8 | Part 3 |  |  | 0.506 | 0.344 |
| item9 | Part 3 |  |  | 0.732 | 0.582 |
| item10 | Part 3 |  |  | 0.834 | 0.705 |
| **Kaiser-Meyer-Olkin Measure of Sampling Adequacy = 0.877** | | | | | |

The boldface indicates salient (> 0.30) loading


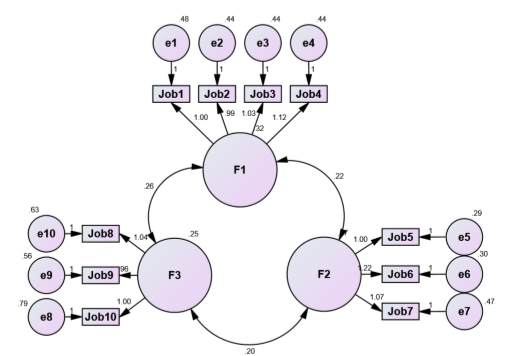


**Figure 2s: CFA confirmatory factor analysis by Structure Equation Modeling for Job Embeddedness scale**

Model fit parameters CFI; IFI; RMSEA (0.952; 0.953; 0.063).

CFI = Comparative fit index; IFI = incremental fit index; and RMSEA = Root Mean Square Error of Approximation.

Model ^^; significance 21.097^*^(0.001*)

**Table 9s: Corrected Item-Total Correlations for Job Embeddedness scale**

| **Item number** | **Section** | **Correlation** |
| --- | --- | --- |
| item1 | Part I | 0.734^*^ |
| item2 | Part I | 0.785^*^ |
| item3 | Part I | 0.748^*^ |
| item4 | Part I | 0.772^*^ |
| item5 | Part 2 | 0.806^*^ |
| item6 | Part 2 | 0.820^*^ |
| item7 | Part 2 | 0.795^*^ |
| item8 | Part 3 | 0.699^*^ |
| item9 | Part 3 | 0.708^*^ |
| item10 | Part 3 | 0.759^*^ |

Correlation: Pearson coefficient *: Statistically significant at p ≤ 0.05

**Table 10s: Correlations between Job Embeddedness scale dimensions**

|  | | **Part 1** | **Part 2** | **Part 3** | **Job Embeddedness scale** |
| --- | --- | --- | --- | --- | --- |
| **Part 1** | **r** |  |  |  |  |
|  | **p** |  |  |  |  |
| **Part 2** | **r** | 0.560^*^ |  |  |  |
|  | **p** | <0.001^*^ |  |  |  |
| **Part 3** | **r** | 0.569^*^ | 0.479^*^ |  |  |
|  | **p** | <0.001^*^ | <0.001^*^ |  |  |
| **Overall Job Embeddedness scale** | **r** | 0.885^*^ | 0.796^*^ | 0.807^*^ |  |
|  | **p** | <0.001^*^ | <0.001^*^ | <0.001^*^ |  |

r: Pearson coefficient *: Statistically significant at p ≤ 0.05

**Table 11s: Cronbach's Alpha for Job Embeddedness scale and its Subscales**

|  | **N of Items** | **Cronbach’s alpha** |
| --- | --- | --- |
| Part 1 | 4 | 0.755 |
| Part 2 | 3 | 0.728 |
| Part 3 | 3 | 0.841 |
| **Job Embeddedness scale** | **10** | **0.834** |
